# Supplementary material for: Genome-wide identification of genes required for alternative peptidoglycan cross-linking in Escherichia coli revealed unexpected impacts of β-lactams
Source: Nat Commun. 2022 Dec 27;13:7962. doi: 10.1038/s41467-022-35528-3 (PMC9794725; doi:10.1038/s41467-022-35528-3)
Supplement: Supplementary file 1 — Supplementary information [file 41467_2022_35528_MOESM1_ESM.pdf]

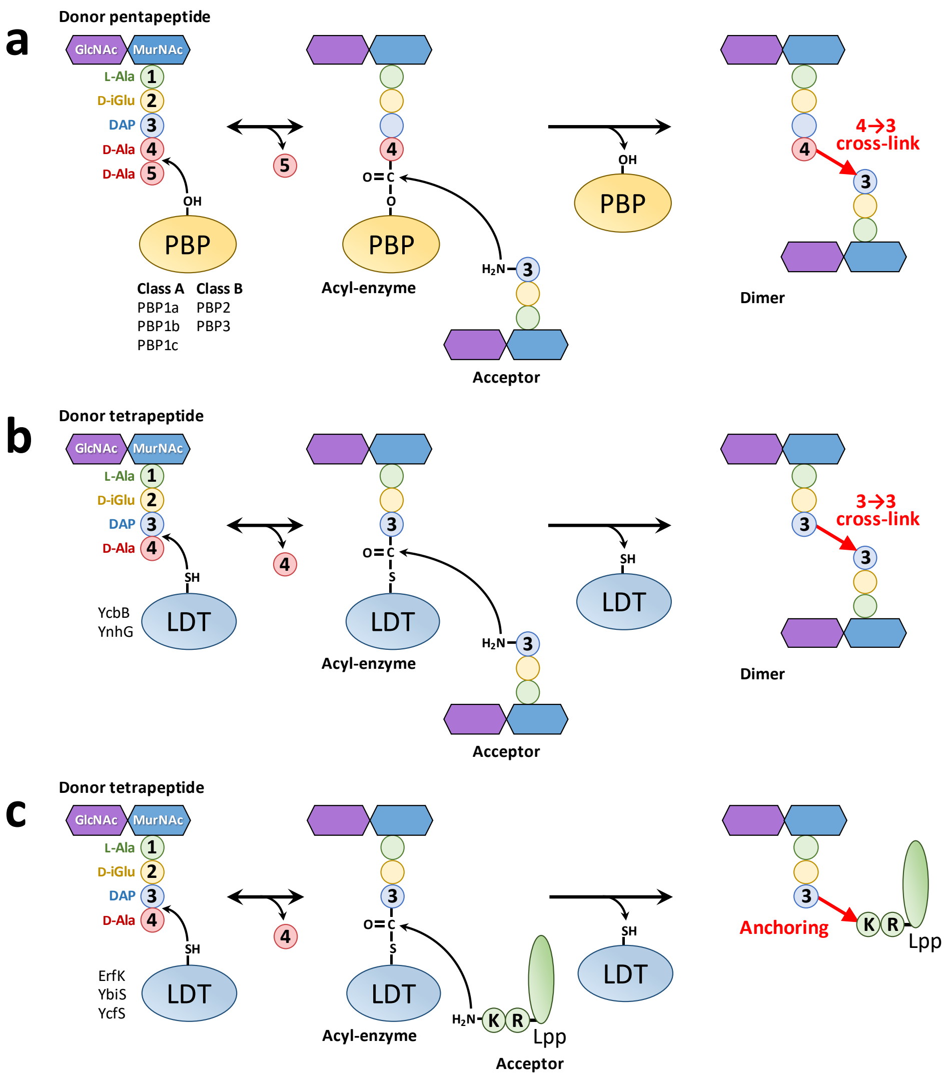

Supplementary Figure S1. Reactions catalyzed by PBPs and L,D-transpeptidases. (**a**) Nucleophilic attack of the carbonyl of D-Ala[4] in a pentapeptide donor stem by the catalytic Ser residue of the D,D-transpeptidase results in the release of D-Ala[5] and formation of an acyl-enzyme. In the following step, nucleophilic attack of the carbonyl of the ester bond by the side-chain amino group of the diaminopimelyl (DAP) residue of an acceptor leads to the release of the PBP and to the formation a peptidoglycan dimer. The corresponding cross-link is referred to as 4→3 as it connects D-Ala at the 4th position of the donor to DAP at the 3rd position of the acceptor. The acceptor stem is a tripeptide or a tetrapeptide resulting in the formation of Tetra→Tri and Tetra→Tetra dimers, respectively. The 4→3 cross-links are potentially formed by three Class A PBPs (PBP1a, PBP1b, PBP1c), which contain glycosyltransferase and D,D-transpeptidase domains, and by two catalytically mono-functional Class B PBPs (PBP2 and PBP3). (**b**) Nucleophilic attack of the carbonyl of DAP[3] in a tetrapeptide donor stem by the catalytic Cys residue of L,D-transpeptidases YcbB and YnhG results in the release of D-Ala[4] and in the formation of an acyl-enzyme. In the following step, nucleophilic attack of the thioester by the DAP residue of the acceptor stem leads to the release of the L,D-transpeptidases and to the formation 3→3 cross-links connecting two DAP residues. (**c**) L,D-transpeptidases ErfK, YbiS, and YcfS anchor the Braun lipoprotein (Lpp) to the peptidoglycan by connecting the 3rd position of a peptidoglycan donor stem to the C-terminal Lys residue of Lpp. The first step involves formation of the acyl-enzyme already described in panel B (for the reaction catalyzed by YcbB and YnhG). In the second step, the thioester bond is attacked by the side-chain amino group of the Lys residue leading to the formation of a Tri→Lpp linkage. For peptidoglycan analysis, treatment of sacculi with trypsin and muramidases leads to the release of a disaccharide-tripeptide substituted by the Lys-Arg residue from the lipoprotein (Tri→KR).

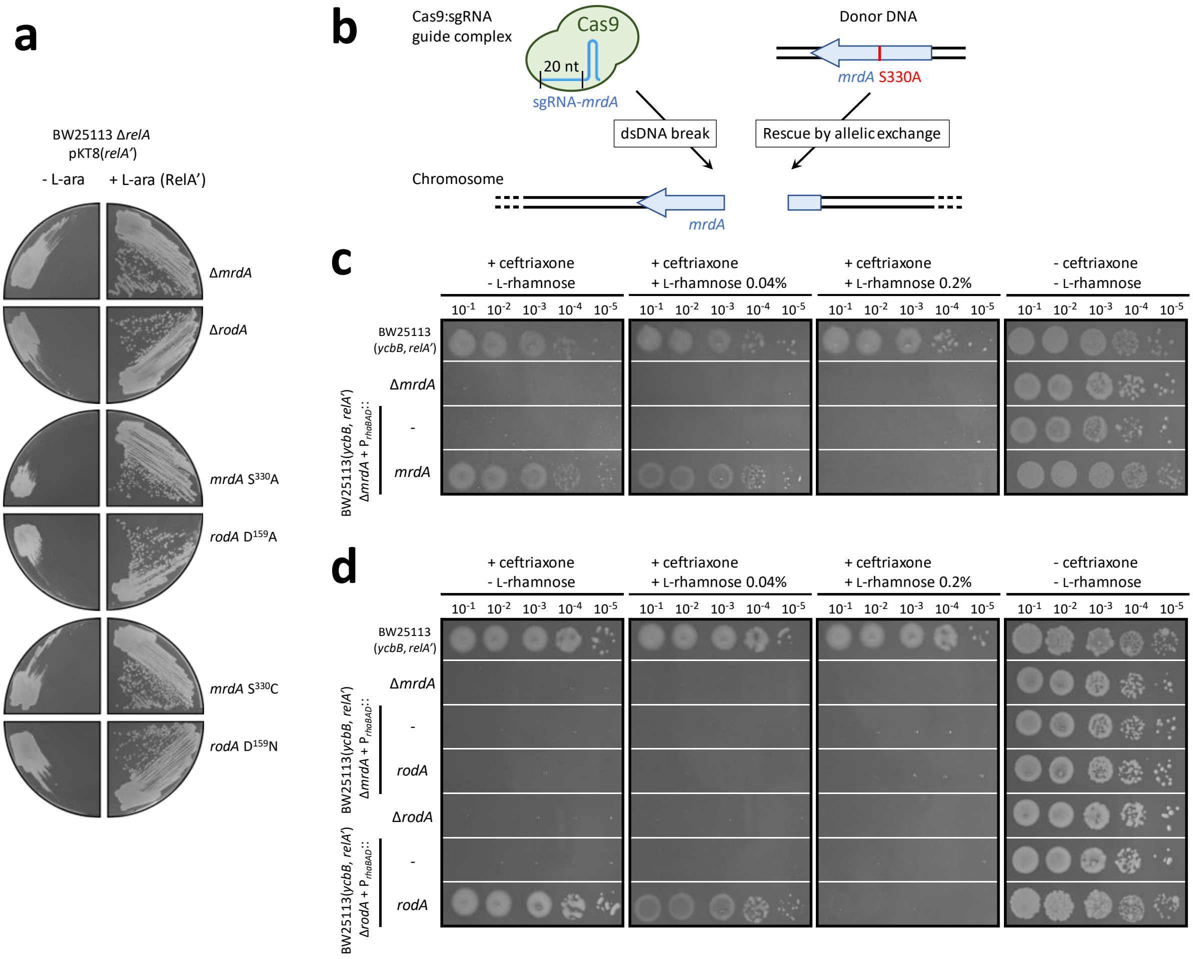

Supplementary Figure S2. Exploring the essentiality of PBP2 and RodA by the CRISPR apporach. (**a**) (p)ppGpp dependence of derivatives of BW25113 Δ*relA* pKT8(*relA'*) harboring mutations in the *mrdA* and *rodA* genes encoding glycosyltransferase RodA and class B PBP PBP2, respectively. The BHI agar plates contained 1% L-arabinose (+L-ara) for induction of *relA'* or no inducer (-L-ara). This analysis showed that growth of *mrdA* and *rodA* mutants is dependent on the expression of the *relA'* (p)ppGpp synthase gene. (**b**) Schematic representation of the approach used to introduce in one step in-frame deletions or point mutations in chromosomal genes encoding PBP2 and RodA. (**c**) Complementation of chromosomal Δ*mrdA* deletion by the expression of the *mrdA* gene expressed under the control of the P_{rhaBAD} promoter of expression vector pHV30. Bacterial growth was tested in the presence of ceftriaxone at 8 µg/ml (+ ceftriaxone) or in the absence of the drug (- ceftriaxone) on BHI agar plates supplemented with 40 µM IPTG and 1% L-arabinose for induction of *ycbB* and *relA'*, respectively. Basal level of expression of *mrdA* in the absence of L-rhamnose was sufficient for complementation. Stronger level of expression of *mrdA* with 0.2% L-rhamnose was toxic. (**d**) Complementation of chromosomal Δ*mrdA* and Δ*rodA* deletions by the expression of the *rodA* gene expressed under the control of the P_{rhaBAD} promoter of expression vector pHV30. Basal level of expression of *rodA* in the absence of L-rhamnose was sufficient for complementation of the *rodA* deletion only. Stronger level of expression of *rodA* with 0.2% L-rhamnose was toxic. These analyses ruled out the possibility that the phenotype associated with the deletion of *mrdA* resulted from a polar effect on the expression of the *rodA* gene.

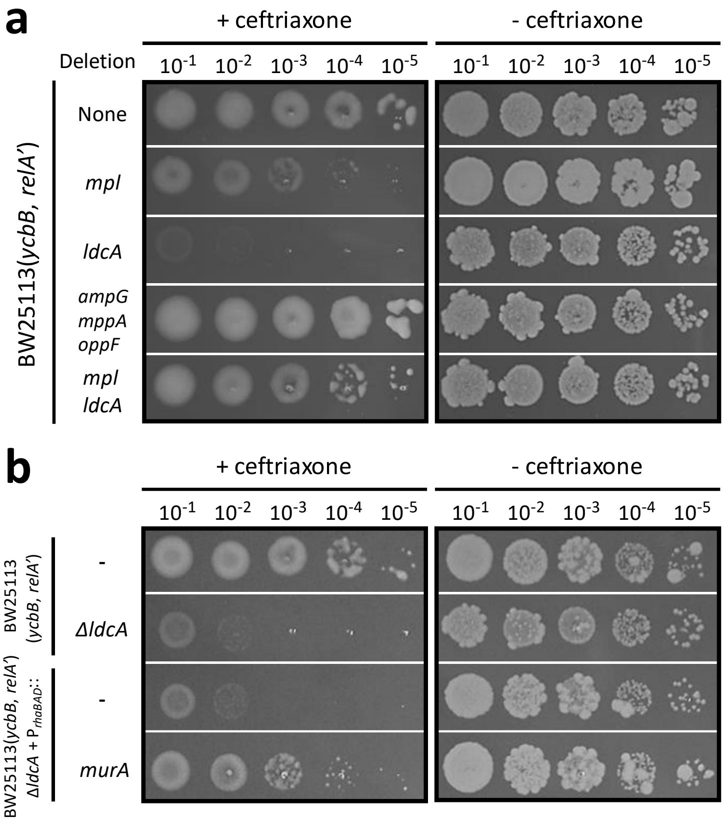

Supplementary Figure S3. Exploring the essentiality of peptidoglycan recycling. (**a**) Bacterial growth was tested in the presence of ceftriaxone at 8 µg/ml (+ ceftriaxone) or in the absence of the drug (- ceftriaxone) on BHI agar plates supplemented with 40 µM IPTG and 1% L-arabinose for induction of *ycbB* and *relA′*, respectively. The triple Δ*ampG*, Δ*mppA*, and Δ*oppF* mutant was deficient in the two permeases for the import of peptidoglycan fragments. In spite of the unessential nature of peptidoglycan recycling, deletion of *ldcA* was not compatible with growth in the presence of ceftriaxone. Resistance of the Δ*ldcA* mutant was restored by the additional deletion of *mpl* encoding the enzyme that ligates tripeptide L-Ala-D-iGlu-DAP and tetrapeptide L-Ala-D-iGlu-DAP-D-Ala onto UDP-MurNAc. Thus, production of UDP-MurNAc-tetrapeptide was responsible for the drug susceptibility of the Δ*ldcA* single mutant. (**b**) Overexpression of the *murA* gene compensated for the inactivation of *ldcA*. The *murA* gene was expressed under the control of the P_{rhaBAD} promoter of vector pHV30 in the presence of 0.2% L-rhamnose.

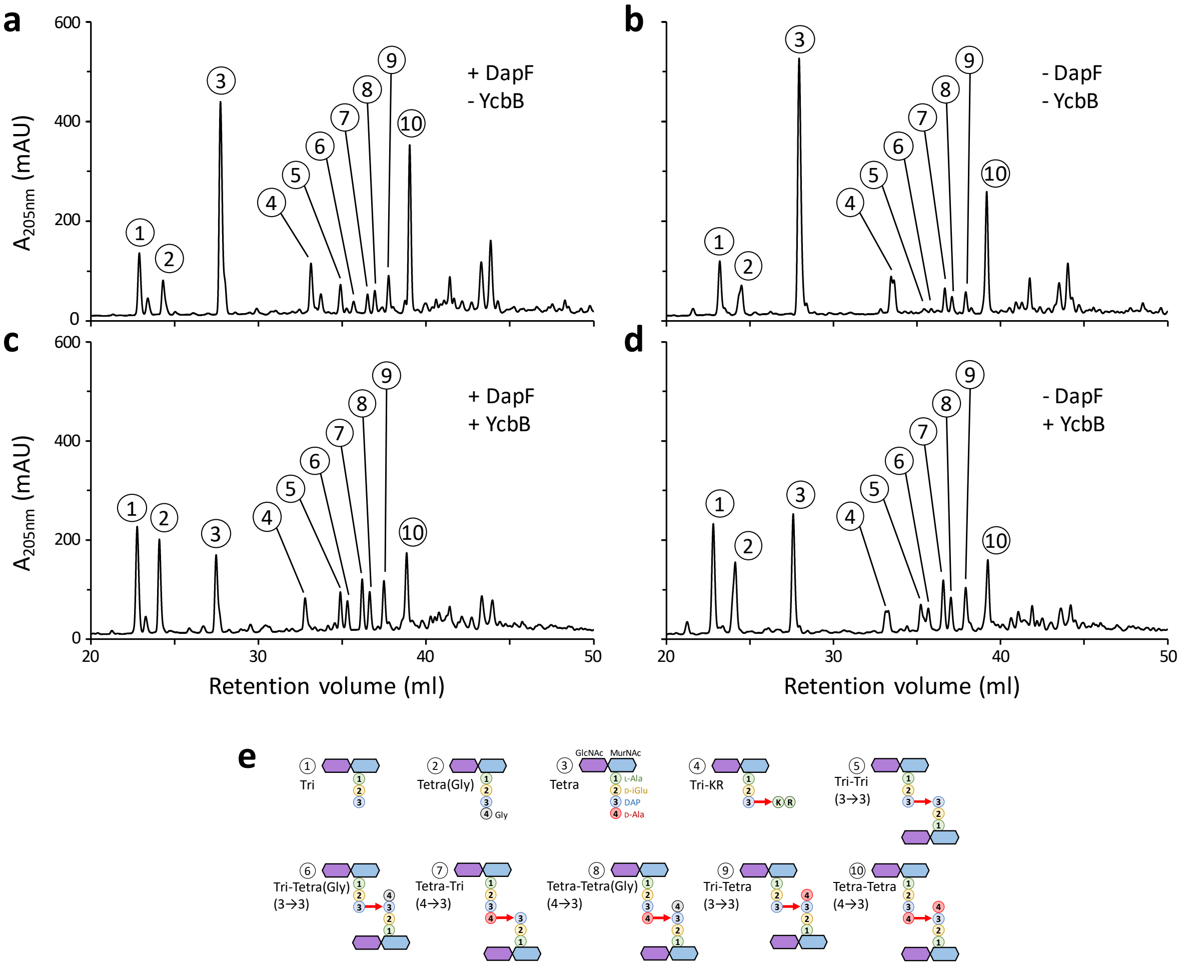

Supplementary Figure S4. Replacement of *meso*DAP by L,L-DAP is compatible with formation of 3→3 cross-links and with the anchoring of the Braun lipoprotein. Peptidoglycan was extracted, digested by muramidases, and the resulting muropeptides were separated by *rp*HPLC (mAU, milli-absorbance unit, λ=205 nm). The structure of muropeptides in *rp*HPLC peaks 1 to 10 was identified by mass spectrometry. (**a** and **b**) Peptidoglycan was extracted from BW25113Δ*relA* harboring vector pHV6 (+DapF -YcbB) and its derivative obtained by deletion of *dapF* (-DapF -YcbB) respectively. (**c** and **d**) Peptidoglycan was extracted from BW25113Δ*relA* harboring plasmid pKT2(*ycbB*) (+DapF +YcbB) and its derivative obtained by deletion of the *dapF* gene (-DapF +YcbB), respectively. Bacteria were grown in the presence of tetracycline [to counter select loss of pHV6 and pKT2(ycbB)] and 40 µM IPTG (for induction of *ycbB*). (**e**) Structure of muropeptides. Tetra, tetrapeptide L-Ala-D-iGlu-DAP-D-Ala; Tri, tripeptide L-Ala-D-iGlu-DAP; Tri-KR, tripeptide L-Ala-D-iGlu-DAP containing the dipeptide Lys-Arg originating from the Braun lipoprotein; Tri-Tri and Tri-Tetra, dimers containing a tripeptide donor stem linked to a tripeptide or tetrapeptide acceptor, respectively. These dimers contained a 3→3 cross-link formed by the YcbB L,D-transpeptidase. Tetra-Tri and Tetra-Tetra, dimers containing a tetrapeptide donor linked to a tripeptide or tetrapeptide acceptor, respectively. These dimers contained a 4→3 cross-link formed by the D,D-transpeptidases belonging to the PBP family; Tetra(Gly), Tri-Tetra(Gly), and Tetra-Tetra(Gly), muropeptides containing a Gly residue instead of D-Ala at the 4th position of the free stem. These muropeptides originated from the exchange of D-Ala by Gly catalyzed by L,D-transpeptidases. Data are representative chromatograms from three biological repeats. Source data are provided as a Source Data file.

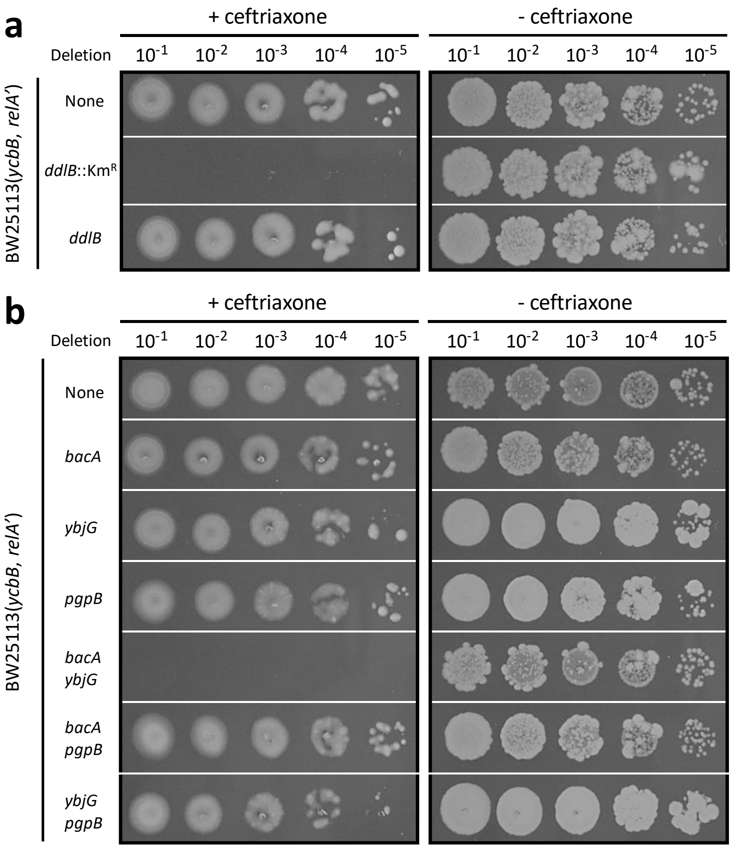

Supplementary Figure S5. Essentiality of genes encoding redundant peptidoglycan biosynthesis enzymes. (**a**) Testing the essentiality of *ddlB* in strain BW25113(*ycbB*, *relA′*) encoding one of the two D-Ala-D-Ala ligases. Growth was tested in the presence of ceftriaxone at 8 µg/ml (+ ceftriaxone) or in the absence of the drug (- ceftriaxone) on BHI agar plates supplemented with 40 µM IPTG and 1% L-arabinose for induction of *ycbB* and *relA′*, respectively. The presence of the Km^R cassette abolished ceftriaxone resistance. Removing the Km^R cassette from the *ddlB* deletion restored ceftriaxone resistance, indicating that *ddlB* is not required for ceftriaxone resistance and that its essentiality detected in the Tn-seq likely results from a polar effect on the downstream *ftsQAZ* and *lpxC* genes. (**b**) Testing the essentiality and redundancy of C_{55}-PP phosphatase encoding genes in strain BW25113(*ycbB*, *relA′*). Growth was tested in the presence of ceftriaxone at 8 µg/ml (+ ceftriaxone) or in the absence of the drug (- ceftriaxone) on BHI agar plates supplemented with 40 µM IPTG and 1% L-arabinose for induction of *ycbB* and *relA′*, respectively. The derivative strain harboring the double deletion of *bacA* and *ybjG* was susceptible to ceftriaxone, indicating that PgpB alone cannot support growth in the presence of the drug.

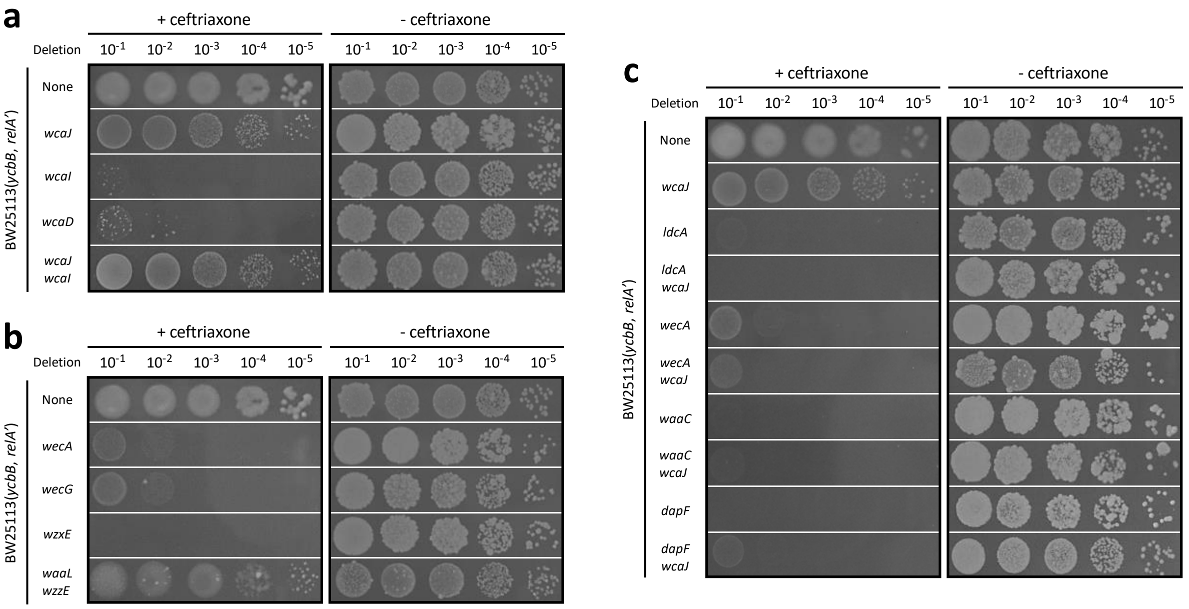

Supplementary Figure S6. Essentiality of genes encoding enzymes for colanic acid and ECA synthesis. Growth of mutants defective in colanic acid synthesis (**a**) and ECA synthesis (**b**) was tested in the presence of ceftriaxone at 8 µg/ml (+ ceftriaxone) or in the absence of the drug (- ceftriaxone) on BHI agar plates supplemented with 40 µM IPTG and 1% L-arabinose for induction of *ycbB* and *relA'*, respectively. (**c**) Additional control experiment showing that the *ldcA*, *wecA*, *waaC*, and *dapF* genes remained essential in the absence of colanic acid synthesis.

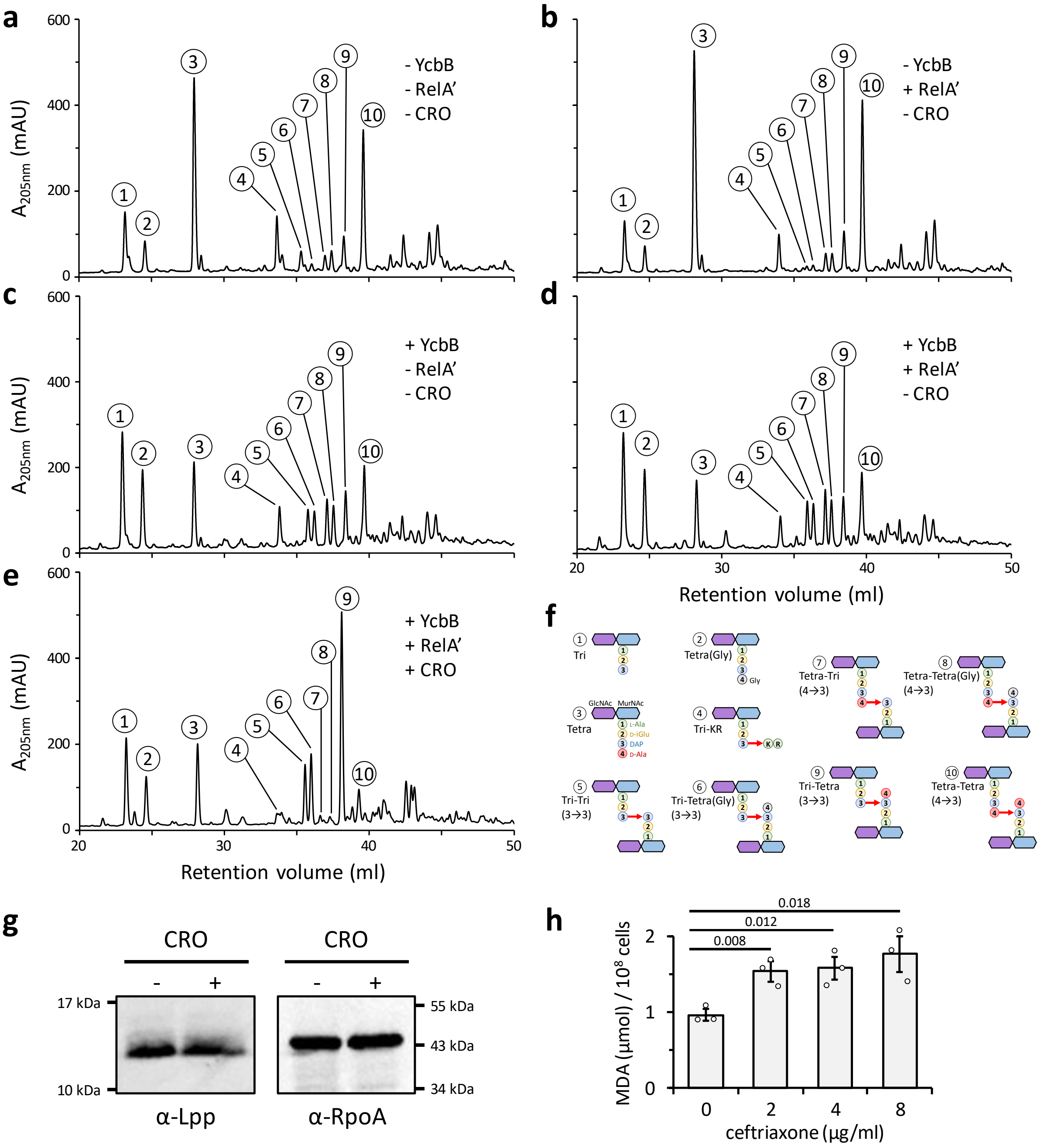

Supplementary Figure S7. Impact of ceftriaxone on the anchoring of the Braun lipoprotein to peptidoglycan. Peptidoglycan was extracted, digested by muramidases, and the resulting muropeptides were separated by *rp*HPLC (mAU, milli-absorbance unit, λ=205 nm). Peptidoglycan was extracted from derivatives of strains BW25113Δ*relA* harboring vectors pHV6 and pHV7 (**a**), vector pHV7 and plasmid pKT2(*ycbB*) (**b**), vector pHV6 and plasmid pKT8(*relA'*) (**c**), and plasmids pKT2(*ycbB*) and pKT8(*relA'*) (**d** and **e** for growth in the absence or presence of ceftriaxone, respectively). The growth medium contained tetracycline (to counter select loss of pHV6 and pKT2), chloramphenicol (to counter select loss of pHV7 and pKT8), except for BW25113Δ*relA* pKT2(*ycbB*) pKT8(*relA'*) grown in the presence of ceftriaxone (as this drug counter select loss of both plasmids). All cultures contained 40 µM IPTG for induction of the P_{trc} promoter of pHV6 and pKT2(*ycbB*) and 1% L-arabinose for induction of the P_{araBAD} promoter of pHV7 and pKT8(*relA'*). The Tri-KR muropeptide (peak 4) was present in all chromatograms but with a much lower abundance for BW25113Δ*relA* pKT2(*ycbB*) pKT8(*relA'*) grown in the presence of ceftriaxone indicating that this drug prevents the anchoring of the Braun lipoprotein to peptidoglycan. (**f**) Structure of the muropeptides deduced from mass spectrometry analyses. Data are

representative chromatograms from three biological repeats. (**g**) Western blot analysis. Crude bacterial extracts of BW25113(*ycbB*, *relA'*) grown in the -CRO and +CRO conditions were separated by SDS-PAGE. Lpp and RpoA were detected with polyclonal antibodies. Images are one representative of three biological repeats. (**h**) Determination of lipid peroxidation. The concentration of malondialdehyde (MDA) was determined spectrophotometrically (λ = 532 nm). Data are the mean and standard deviation from three biological repeats. *p*-values were obtained from unpaired two-tailed *t*-tests. Source data are provided as a Source Data file.

Table S1. Characteristics and origin of the strains, plasmids and oligonucleotides used in this study

**Strain**	**Characteristics**				**Origin**
BW25113	Δ(*araD-araB*)*567* Δ(*rhaD-rhaB*)*568* Δ*lacZ4787* (::*rrnB-3*) *hsdR514 rph-1*				Baba, T. *et al* [58]
BW25113(*ycbB*, *relA′*)	Δ*relA* derivative of BW25113 harboring pKT2(*ycbB*) and pKT8(*relA′*) plasmids				Hugonnet, J. E. *et al* [8]
**Plasmid**	**Characteristics**				**Origin**
Vectors					
pHV6	Tet^R	P_{trc}	*lacI*	*oriV* CloDF13	Voedts, H. *et al* [7]
pHV7	Cm^R	P_{araBAD}	*araC*	*oriV* p15A	Voedts, H. *et al* [7]
pHV30	Zeo^R	P_{rhaBAD}	*rhaSR*	*oriV* pSC101	Voedts, H. *et al* [7]
Recombinant plasmids for ycbB and relA′ expression					
pKT2	pHV6Ω*ycbB*				Hugonnet, J. E. *et al* [8]
pKT8	pHV7Ω*relA′*				Hugonnet, J. E. *et al* [8]
Recombinant plasmids for complementation of rodA and ldcA gene deletions					
pHV112	pHV30Ω*murA*				This study
pHV113	pHV30Ω*rodA*				This study
pHV131	pHV30Ω*mrdA*				This study
Recombinant plasmids for CRISPR-Cas9 mutagenesis					
pCas	Km^R	P_{araBAD} λ Red	*araC*	*cas9*	Jiang, Y. *et al* [61]
		P_{trc} *sgRNA*-pMB1	*lacI*	*oriV* $pSC101^{ts}$	
pTargetF	Sm^R	*sgRNA*		*oriV* pMB1	Jiang, Y. *et al* [61]
pTargetF-*mrdA*	Sm^R	*sgRNA-mrdA*		*oriV* pMB1	This study
pTargetF-*rodA*	Sm^R	*sgRNA-rodA*		*oriV* pMB1	This study
**Oligonucleotide**	**Sequence (5′→3′)**				
Junction fragment amplification					
HV1	CAAGCAGAAGACGGCATACGAGATNNNNNNGTGACTGGAGTTCAGACGTGTGCTCTTCCGATCTGAGACACAATTCATCGATGATG				
HV2	CAAGCAGAAGACGGCATACGAGATNNNNNNGTGACTGGAGTTCAGACGTGTGCTCTTCCGATCTGACCTGCAGGCATGCAAGC				
pTargetF-mrdA/rodA construction					
HV3	AAACCCTATGGTTTTAGAGCTAGAAATAGC				
HV4	AACTGTAGACACTAGTATTATACCTAGGAC				
HV5	CAGCCTGACCGTTTTAGAGCTAGAAATAGC				
HV6	TGCAGCCACCACTAGTATTATACCTAGGAC				
mrdA S330A/C donor DNA amplification					
HV7	ATGAAACTACAGAACTCTTTTCG				
HV8	TTAATGGTCCTCCGCTGCGG				
rodA D159A/N donor DNA amplification					
HV9	ATGACGGATAATCCGAATAAAAAAAC				
HV10	TTACACGCTTTTCGACAACATTTTC				
ΔmrdA donor DNA amplification					
HV11	GTTCGCGTGCTGGTCGCAG				
HV12	ATAACCAGGGCGCTGTAAACC				
ΔrodA donor DNA amplification					
HV13	CCTCGACCACATTATGCTGGG				
HV14	TTGTACATGCCGCGAGCATTC				
