# Supplementary material for: Genome-wide identification of genes required for alternative peptidoglycan cross-linking in Escherichia coli revealed unexpected impacts of β-lactams
Source: Nat Commun. 2022 Dec 27;13:7962. doi: 10.1038/s41467-022-35528-3 (PMC9794725; doi:10.1038/s41467-022-35528-3)
Supplement: Supplementary file 3 — Description to Additional Supplementary Information [file 41467_2022_35528_MOESM3_ESM.pdf]

## Description of Supplementary Data files

**File Name: Supplementary Data file 1.** (**a**) Tn-seq data set. (**b**) List of the 179 genes essential for the +CRO condition.

**File Name: Supplementary Data file 2.** Tn-seq insertion profiles of genes highlighted in the main text. Reading frames are indicated at the bottom of the panels and color-coded: red, genes selectively essential for the +CRO condition; yellow, genes for which inactivation incurred a fitness cost for +CRO; green, genes essential for both -CRO and +CRO; gray, genes unessential in either condition. Transposon insertion sites are indicated by lines above the reading frames with their height reflecting the number of reads for each insertion.
