# Supplementary material for: Genome-wide identification of genes required for alternative peptidoglycan cross-linking in Escherichia coli revealed unexpected impacts of β-lactams
Source: Nat Commun. 2022 Dec 27;13:7962. doi: 10.1038/s41467-022-35528-3 (PMC9794725; doi:10.1038/s41467-022-35528-3)
Supplement: Supplementary file 5 — Supplementary Dataset 2 [file 41467_2022_35528_MOESM5_ESM.pdf]

Supplementary Data file 2

Tn-seq insertion profiles of relevant genes discussed in the manuscript. Reading frames are indicated at the bottom of the panel and color coded: gray, genes unessential in either condition; green, genes essential for both -CRO and +CRO; red, genes selectively essential for +CRO; yellow, genes for which inactivation induces a reduced fitness for +CRO. Transposon insertion sites are indicated by lines above the reading frames with their height reflecting the number of reads for each insertion.

Color code:

- non-essential (-CRO and +CRO)
- essential +CRO
- essential (-CRO and +CRO)
- reduced fitness +CRO

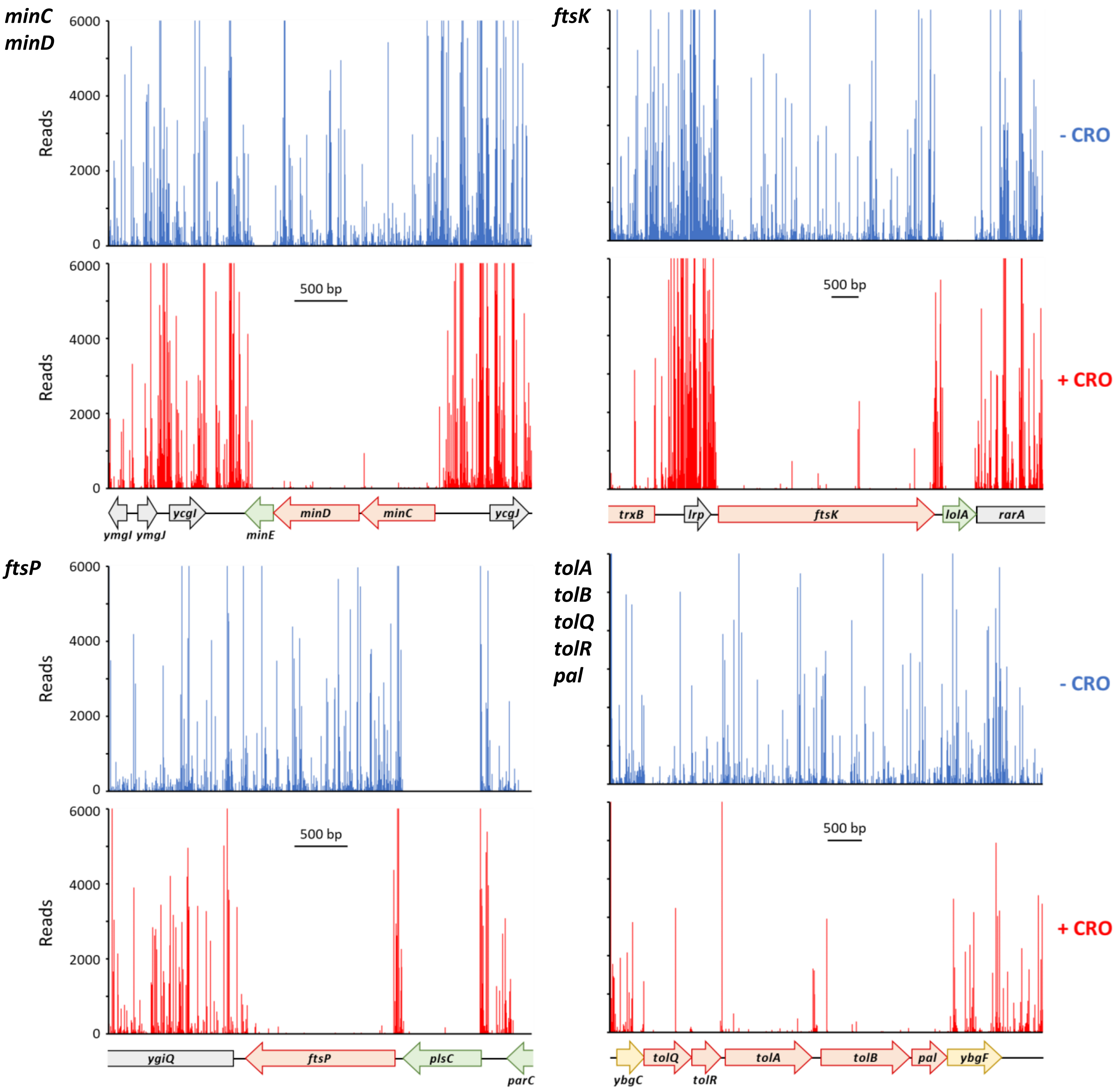

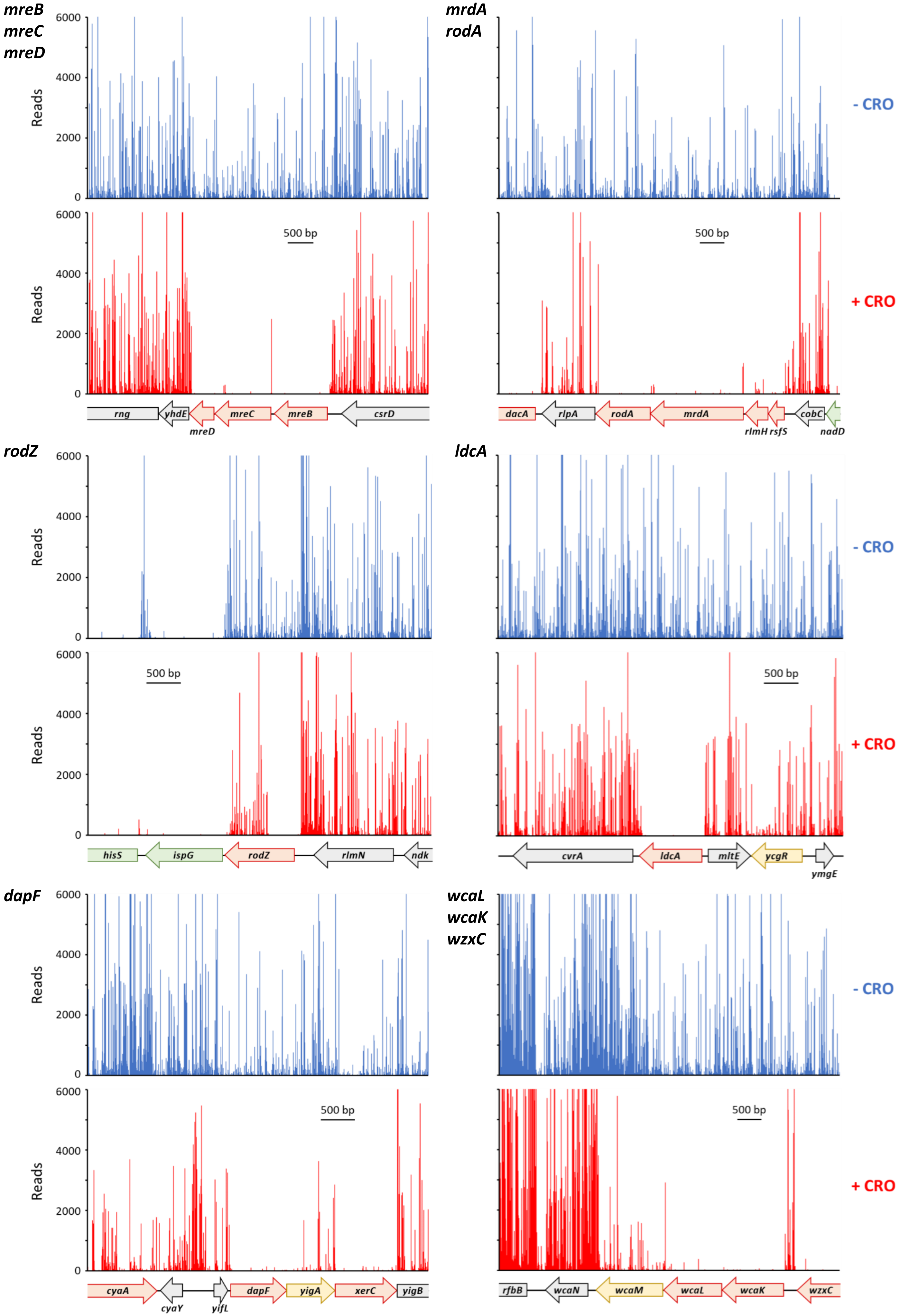
mreB
mreC
mreD
mrdA
rodA
rodZ
ldcA
dapF
wcaL
wcaK
wzxC
Reads
6000
4000
2000
0
500 bp
- CRO
+ CRO
rng
yhdE
mreD
mreC
mreB
csrD
dacA
rlpA
rodA
mrdA
rlmH
rsfS
cobC
nadD
hisS
ispG
rodZ
rlmN
ndk
cvrA
ldcA
mltE
ycgR
ymgE
cyaA
cyaY
yifL
dapF
yigA
xerC
yigB
rfbB
wcaN
wcaM
wcaL
wcaK
wzxC

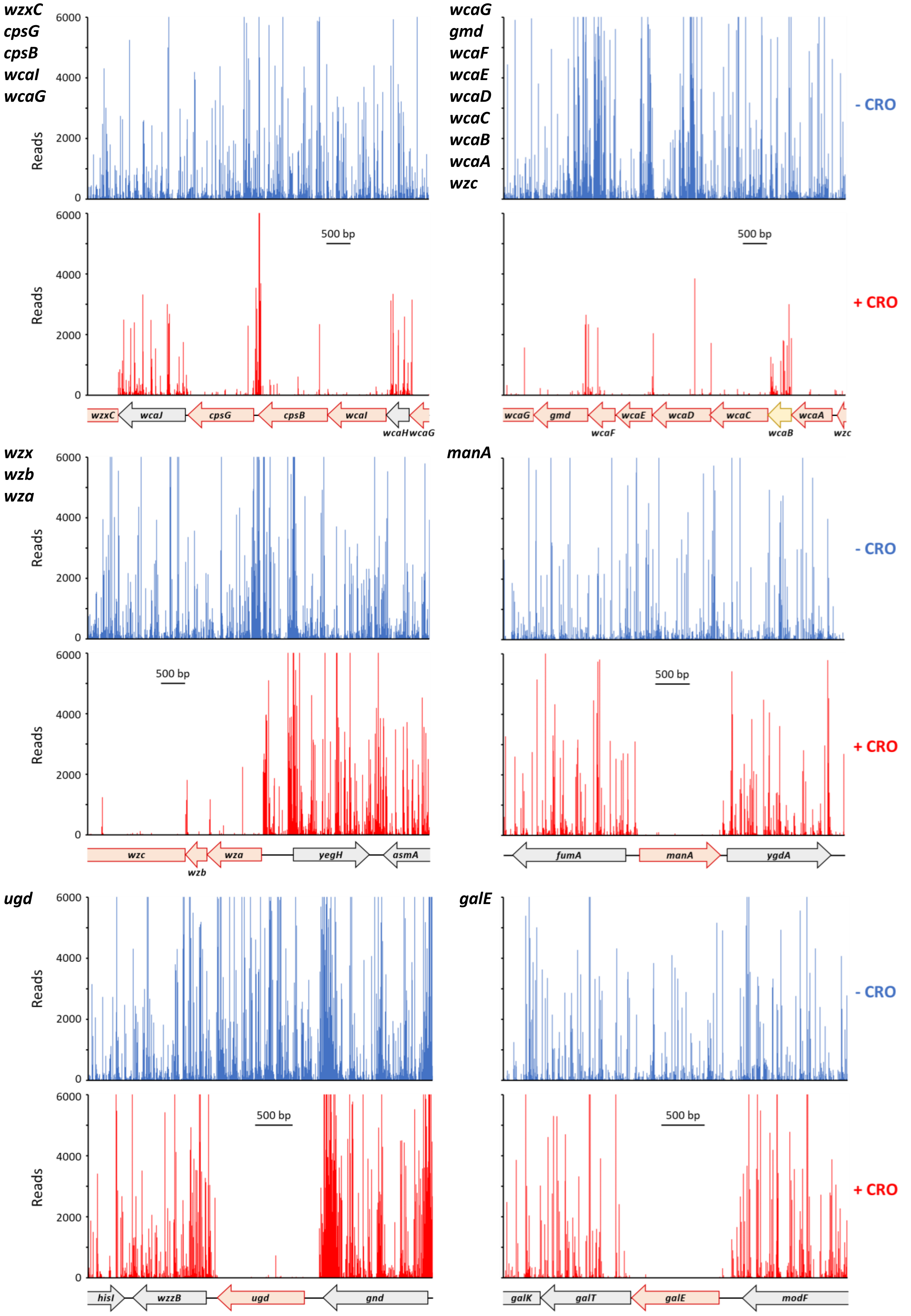
wzxC
cpsG
cpsB
wcaI
wcaG
wcaG
gmd
wcaF
wcaE
wcaD
wcaC
wcaB
wcaA
wzc
- CRO
+ CRO
Reads
6000
4000
2000
0
500 bp
wzxC
wcaJ
cpsG
cpsB
wcaI
wcaH
wcaG
wcaG
gmd
wcaF
wcaE
wcaD
wcaC
wcaB
wcaA
wzc
wzx
wzb
wza
manA
- CRO
+ CRO
Reads
500 bp
wzc
wzb
wza
yegH
asmA
fumA
manA
ygdA
ugd
galE
- CRO
+ CRO
Reads
500 bp
hisI
wzzB
ugd
gnd
galK
galT
galE
modF

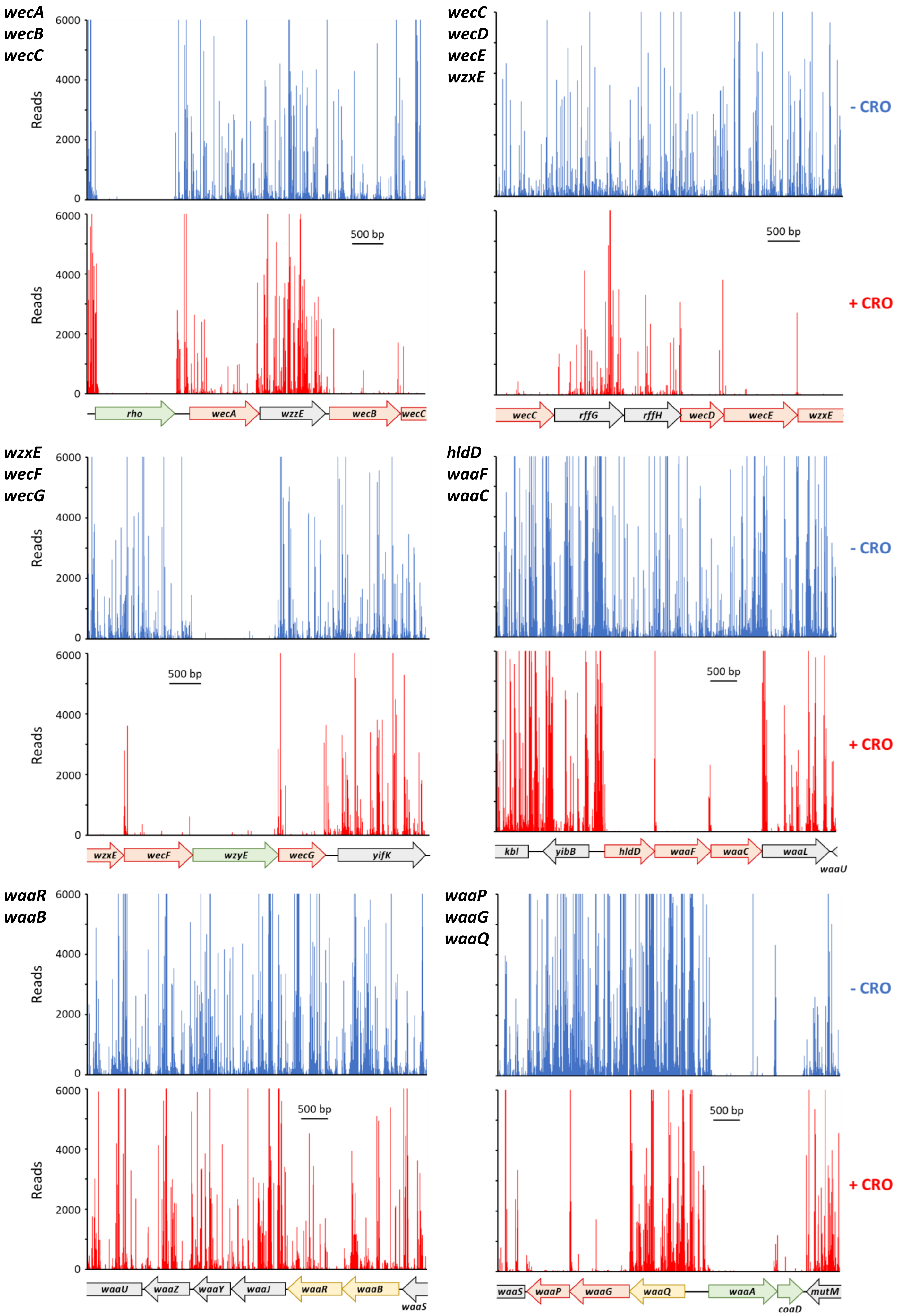
wecA
wecB
wecC
wecC
wecD
wecE
wzxE
- CRO
+ CRO
Reads
6000
4000
2000
0
500 bp
rho
wecA
wzzE
wecB
wecC
wecC
rffG
rffH
wecD
wecE
wzxE
wzxE
wecF
wecG
hldD
waaF
waaC
wzxE
wecF
wzyE
wecG
yifK
kbl
yibB
hldD
waaF
waaC
waaL
waaU
waaR
waaB
waaP
waaG
waaQ
waaU
waaZ
waaY
waaJ
waaR
waaB
waaS
waaS
waaP
waaG
waaQ
waaA
coaD
mutM

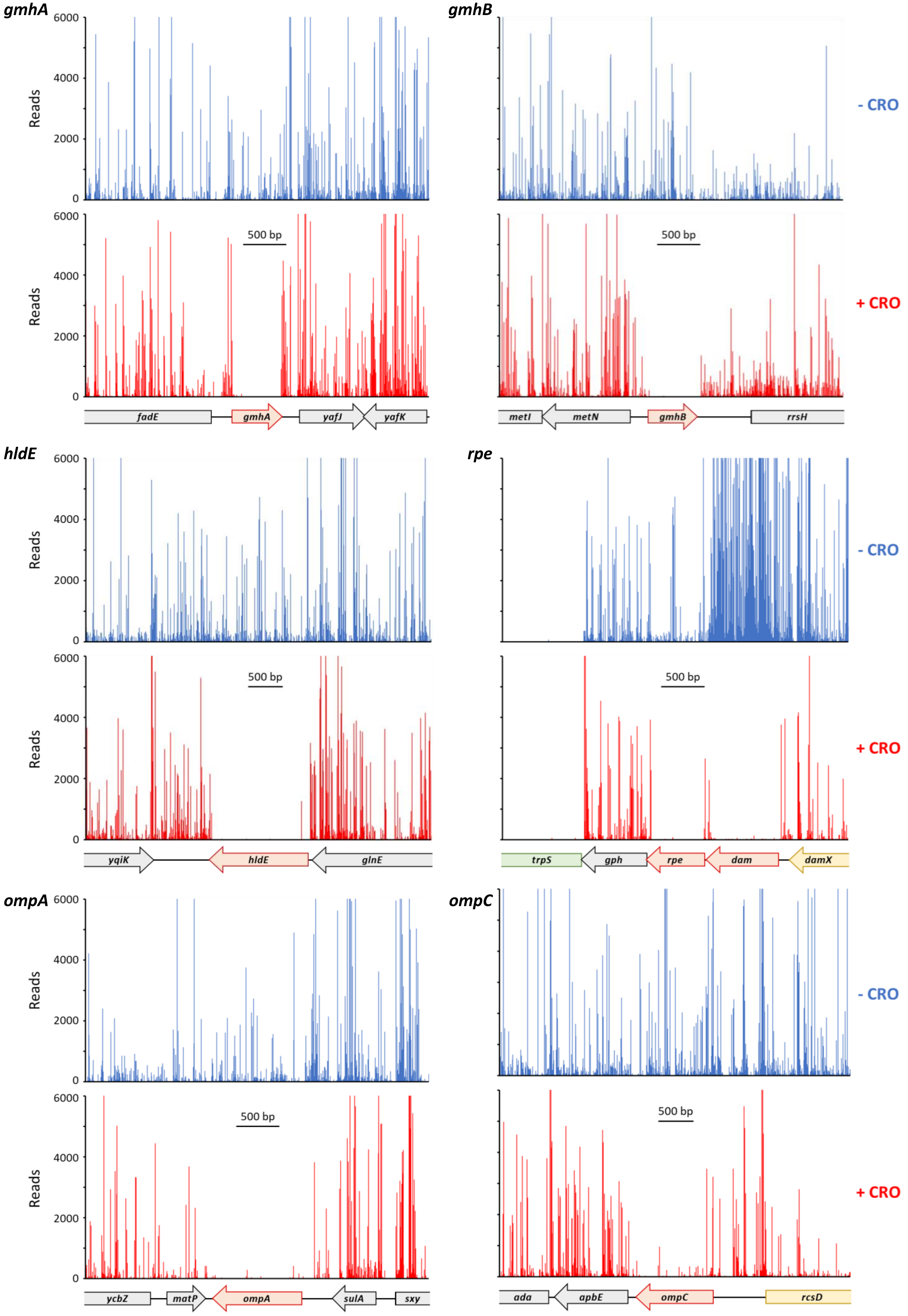
gmhA
gmhB
hldE
rpe
ompA
ompC
Reads
6000
4000
2000
0
- CRO
+ CRO
500 bp
fadE
gmhA
yafJ
yafK
metI
metN
gmhB
rrsH
yqiK
hldE
glnE
trpS
gph
rpe
dam
damX
ycbZ
matP
ompA
sulA
sxy
ada
apbE
ompC
rcsD
