# Supplementary material for: Genome-wide identification of genes required for alternative peptidoglycan cross-linking in Escherichia coli revealed unexpected impacts of β-lactams
Source: Nat Commun. 2022 Dec 27;13:7962. doi: 10.1038/s41467-022-35528-3 (PMC9794725; doi:10.1038/s41467-022-35528-3)
Supplement: Supplementary file 6 — Reporting Summary [file 41467_2022_35528_MOESM6_ESM.pdf]

nature portfolio

Corresponding author(s): Jean-Emmanuel Hugonnet

Last updated by author(s): Dec 1, 2022

Reporting Summary

Nature Portfolio wishes to improve the reproducibility of the work that we publish. This form provides structure for consistency and transparency in reporting. For further information on Nature Portfolio policies, see our Editorial Policies and the Editorial Policy Checklist.

Statistics

For all statistical analyses, confirm that the following items are present in the figure legend, table legend, main text, or Methods section.

n/a	Confirmed	
☐	☒	The exact sample size (*n*) for each experimental group/condition, given as a discrete number and unit of measurement
☐	☒	A statement on whether measurements were taken from distinct samples or whether the same sample was measured repeatedly
☐	☒	The statistical test(s) used AND whether they are one- or two-sided<br>*Only common tests should be described solely by name; describe more complex techniques in the Methods section.*
☒	☐	A description of all covariates tested
☒	☐	A description of any assumptions or corrections, such as tests of normality and adjustment for multiple comparisons
☐	☒	A full description of the statistical parameters including central tendency (e.g. means) or other basic estimates (e.g. regression coefficient) AND variation (e.g. standard deviation) or associated estimates of uncertainty (e.g. confidence intervals)
☐	☒	For null hypothesis testing, the test statistic (e.g. *F*, *t*, *r*) with confidence intervals, effect sizes, degrees of freedom and *P* value noted<br>*Give P values as exact values whenever suitable.*
☒	☐	For Bayesian analysis, information on the choice of priors and Markov chain Monte Carlo settings
☒	☐	For hierarchical and complex designs, identification of the appropriate level for tests and full reporting of outcomes
☒	☐	Estimates of effect sizes (e.g. Cohen's *d*, Pearson's *r*), indicating how they were calculated

Our web collection on statistics for biologists contains articles on many of the points above.

Software and code

Policy information about availability of computer code

Data collection	No software was used for data collection.
Data analysis	The Tn-Seq analysis was performed using the Python (v.3.7) package TRANSIT (v.3.0+). Mapping was done using BWA (v.0.7.17). Mass spectra were analyzed using mineXpert2 (v.8.5.2).

For manuscripts utilizing custom algorithms or software that are central to the research but not yet described in published literature, software must be made available to editors and reviewers. We strongly encourage code deposition in a community repository (e.g. GitHub). See the Nature Portfolio guidelines for submitting code & software for further information.

Data

Policy information about availability of data

All manuscripts must include a data availability statement. This statement should provide the following information, where applicable:

- Accession codes, unique identifiers, or web links for publicly available datasets
- A description of any restrictions on data availability
- For clinical datasets or third party data, please ensure that the statement adheres to our policy

The raw Tn-seq data are publicly available at the Sequence Read Archive database (SRA) under the BIOPROJECT accession number PRJNA907050 (https://www.ncbi.nlm.nih.gov/Traces/study/?acc=PRJNA907050).

Human research participants

Policy information about studies involving human research participants and Sex and Gender in Research.

Reporting on sex and gender	Not applicable
Population characteristics	Not applicable
Recruitment	Not applicable
Ethics oversight	Not applicable

Note that full information on the approval of the study protocol must also be provided in the manuscript.

Field-specific reporting

Please select the one below that is the best fit for your research. If you are not sure, read the appropriate sections before making your selection.

☒ Life sciences ☐ Behavioural & social sciences ☐ Ecological, evolutionary & environmental sciences

For a reference copy of the document with all sections, see nature.com/documents/nr-reporting-summary-flat.pdf

Life sciences study design

All studies must disclose on these points even when the disclosure is negative.

Sample size	Sample size calculation was performed for the Tn-seq analysis as follows: the number of independent mutants in the library must represent at least 20 insertions per genes (total of 80,000 clones) to ensure sufficient coverage of the genome. The number of independent insertion events was 810,000 (200 insertions per gene) for the -CRO condition and 260,000 for the +CRO condition (65 insertions per gene).
Data exclusions	No data were excluded from this study.
Replication	Technical duplicates for the Tn-seq analysis were performed and the correlation between the two duplicates was confirmed (Fig. 2a and 2b). For other experiments, all attempts at replication (duplicates or triplicates) were successful.
Randomization	Randomization was not relevant to our study design.
Blinding	Blinding was performed during the sequencing procedure, and not relevant to our study otherwise.

Reporting for specific materials, systems and methods

We require information from authors about some types of materials, experimental systems and methods used in many studies. Here, indicate whether each material, system or method listed is relevant to your study. If you are not sure if a list item applies to your research, read the appropriate section before selecting a response.

Materials & experimental systems

n/a	Involved in the study	
☐	☒	Antibodies
☒	☐	Eukaryotic cell lines
☒	☐	Palaeontology and archaeology
☒	☐	Animals and other organisms
☒	☐	Clinical data
☒	☐	Dual use research of concern

Methods

n/a	Involved in the study	
☒	☐	ChIP-seq
☒	☐	Flow cytometry
☒	☐	MRI-based neuroimaging

Antibodies

Antibodies used	Primary antibodies were obtained from academic collaborators and are not commercially available: Polyclonal rabbit anti-Lpp were provided by Jean-François Collet (De Duve Institute). Polyclonal mouse anti-RpoA were provided by Christophe Beloin (Pasteur Institute).<br>For the secondary antibodies: Anti-Rabbit IgG (whole molecule)–Peroxidase antibody produced in goat, Provider: Sigma-Aldrich, cat

number: A0545-1ML. Anti-Mouse IgG (whole molecule)–Peroxidase antibody produced in goat, Provider: Sigma-Aldrich, cat number: A4416-1ML

Validation

Primary polyclonal rabbit anti-Lpp antibodies were validated in Winkle M. et al, Mbio Vol 12 N°3, 2021. doi: 10.1128/mBio.00836-21
Primary polyclonal mouse anti-RpoA were validated in Chekli Y. et al, Sci Rep Vol 10 (1):15791, 2020. doi: 10.1038/s41598-020-72498-2.
