# Supplementary figures and images for: Genome-wide identification of genes required for alternative peptidoglycan cross-linking in Escherichia coli revealed unexpected impacts of β-lactams
Source: Nat Commun. 2022 Dec 27;13:7962. doi: 10.1038/s41467-022-35528-3 (PMC9794725; doi:10.1038/s41467-022-35528-3)

L -CRO +CRO

L -CRO +CRO

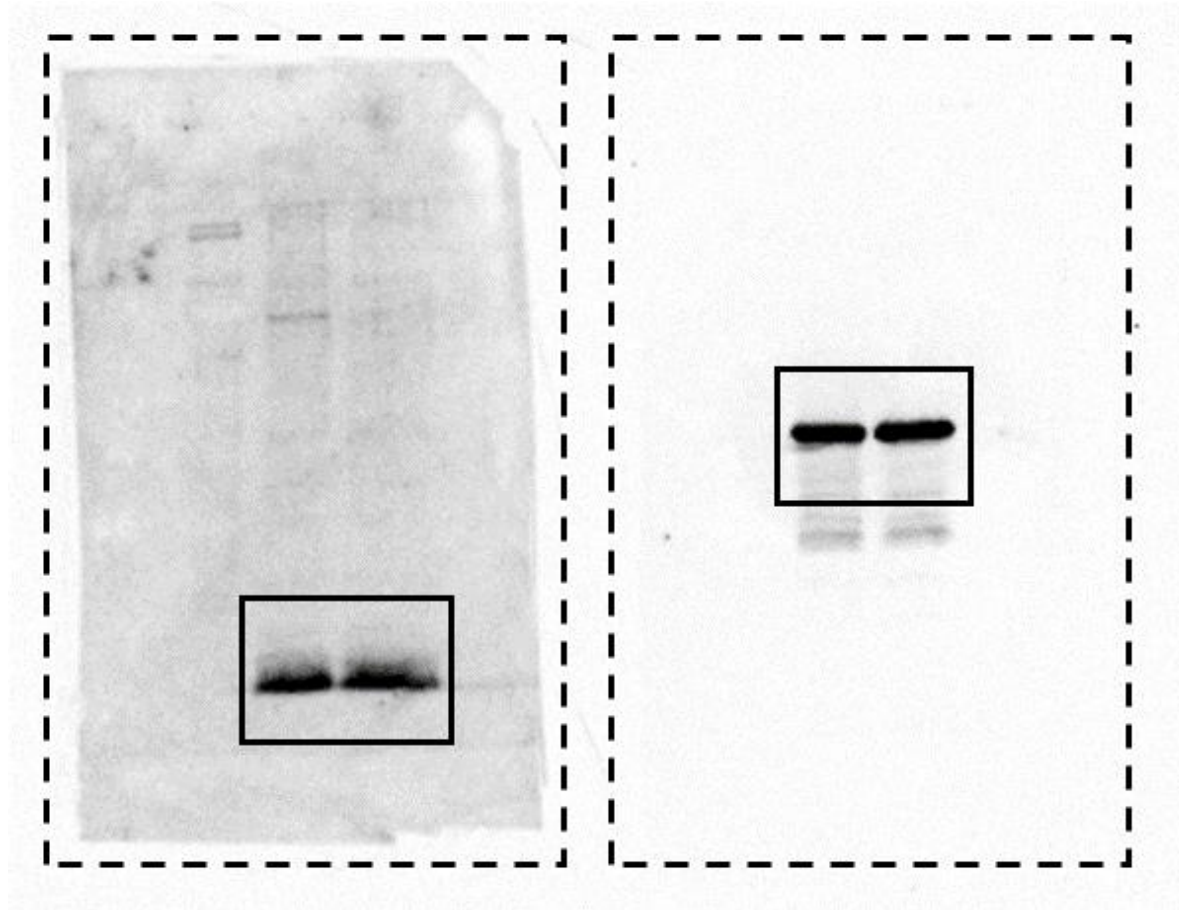

$\alpha$ -Lpp

$\alpha$ -RpoA

Supplement: Supplementary file 7 — Source Data [file 41467_2022_35528_MOESM7_ESM.zip › Source data/NCOMMS-22-32552A - Voedts et al - Source Data Uncropped blots.pdf]
